# Identification of *ARF* Genes and Elucidation of the Regulatory Effects of PsARF16a on the Dormancy of Tree Peony Plantlets

**DOI:** 10.3390/genes15060666

**Published:** 2024-05-23

**Authors:** Zhenzhu Fu, Xin Yuan, Yinge Zhao, Xiaohui Wang, Lin Lu, Huijuan Wang, Yanmin Li, Jie Gao, Limin Wang, Hechen Zhang

**Affiliations:** 1 Horticultural Research Institute, Henan Academy of Agricultural Sciences, Zhengzhou 450002, China; 2 Luoyang Academy of Agriculture and Forestry Sciences, Luoyang 471022, China

**Keywords:** tree peony, dormancy, hormone, PsARF

## Abstract

The low survival rate of transplanted plantlets, which has limited the utility of tissue-culture-based methods for the rapid propagation of tree peonies, is due to plantlet dormancy after rooting. We previously determined that the auxin response factor PsARF may be a key regulator of tree peony dormancy. To clarify the mechanism mediating tree peony plantlet dormancy, *PsARF* genes were systematically identified and analyzed. Additionally, *PsARF16a* was transiently expressed in the leaves of tree peony plantlets to examine its regulatory effects on a downstream gene network. Nineteen *PsARF* genes were identified and divided into four classes. All *PsARF* genes encoded proteins with conserved B3 and ARF domains. The number of motifs, exons, and introns varied between *PsARF* genes in different classes. The overexpression of *PsARF16a* altered the expression of *NCED*, *ZEP*, *PYL*, *GA2ox1*, *GID1*, and other key genes in abscisic acid (ABA) and gibberellin (GA) signal transduction pathways, thereby promoting ABA synthesis and decreasing GA synthesis. Significant changes to the expression of some key genes contributing to starch and sugar metabolism (e.g., *AMY2A*, *BAM3*, *BGLU*, *STP*, and *SUS2*) may be associated with the gradual conversion of sugar into starch. This study provides important insights into *PsARF* functions in tree peonies.

## 1. Introduction

The tree peony (*Paeonia suffruticosa* Andr.) is a famous traditional flower in China, known as the “national beauty” and “the king of flowers.” In recent years, the value of its oil and ornamental features (i.e., as a fresh-cut flower species) and its health benefits have gradually been revealed, making the tree peony an increasingly important species in the floral industry, with a growing market demand [1,2]. Traditionally, tree peonies were propagated from seeds or by plant division and grafting, but the associated methods have drawbacks, such as a long cycle, low reproduction efficiency, and separation of offspring traits [3]. Accordingly, there is a lack of efficient methods for developing improved tree peony varieties and seedlings to satisfy the increasing market demand, which has seriously restricted the industrial development of tree peonies. The short cycle and high reproduction efficiency of tissue culture are conducive to the rapid development of enhanced tree peony varieties. Although a few tree peony plants have been obtained through tissue culture, the current methods must be optimized before they can be applied on an industrial scale. More specifically, the apical buds of plantlets become dormant after rooting, which results in a low survival rate after transplanting [4,5,6].

We previously conducted substantial research on the mechanism underlying tree peony plantlet dormancy. In particular, we measured endogenous hormone contents and performed transcriptome and targeted metabolome analyses during the dormancy process of plantlets. We determined that tree peony plantlet dormancy is closely related to abscisic acid (ABA) and auxin contents and proportions [5]. Aux/IAA and auxin response factor (ARF) transcription factors are key regulators of the auxin signaling pathway [7,8]. On the basis of association analyses, ARF was revealed to be a potentially important regulator of tree peony plantlet dormancy [5]. We speculated that the accumulation of auxin in plantlets after rooting is initiated promotes ABA synthesis, eventually leading to dormancy. Previous studies have shown that auxin and ABA synergistically regulate seed dormancy, with the associated physiological effects caused by changes to ABA signaling pathways [9]. Auxin and ABA contents and signal transduction are reportedly positively correlated with *Arabidopsis thaliana* seed dormancy [10]. Auxin may contribute to ABA-mediated seed dormancy and germination in two ways (i.e., promoting ABA biosynthesis or activating ABA responses) [11]. ARF transcription factors are important for regulating auxin signaling [12]. The *ABI3*, *ABI4*, and *ABI5* genes encode three crucial downstream components of the ABA signal transduction pathway, of which ABI3 is the main regulator of seed dormancy and germination [13]. The inductive effect of auxin on seed dormancy depends on ABI3. Earlier research indicated ARF transcription factors may recruit or activate other specific transcription factors to promote *ABI3* expression, thereby promoting dormancy [9,11]. Among the 23 *AtARF* genes in *A. thaliana* [14], *AtARF10*, *AtARF16*, and *AtARF17* encode important transcription factors affecting the auxin signaling pathway during auxin-induced dormancy [15,16,17]. Both *AtARF10* and *AtARF16* are expressed when auxin levels are high, which leads to the activation of *ABI3* transcription and the maintenance of seed dormancy in *A. thaliana* [9]. In wheat, TaARF15-A1 may down-regulate the expression of some genes that induce leaf senescence, while up-regulating the expression of genes that delay senescence, ultimately promoting dormancy [18]. Auxin and ARF10/16 are required for the maintenance of *ABI3* expression in seeds. Moreover, ABI3, AtARF10/16, and bZIP67 are transcription factors that bind to RY and GBL elements in the *DOG1* promoter region to regulate *A. thaliana* seed dormancy and germination [19]. Therefore, auxin signaling pathways are necessary for plant dormancy, with AtARF10 and AtARF16 functioning as key downstream components.

The completely sequenced tree peony genome [20,21] is useful for identifying and functionally characterizing tree peony genes. However, there are currently no reports of studies on tree peony *PsARF* genes. In the current study, tree peony *PsARF* gene family members were systematically identified on the basis of genome data and full-length transcriptome sequencing data. In addition, we cloned *PsARF16a*, which is a homolog of the *A. thaliana* gene (*AtARF16*) associated with dormancy, and overexpressed it in tree peonies using a transient expression system to explore its regulatory effects on a downstream gene network. The results preliminarily revealed the regulatory effect of PsARF16a on ABA synthesis, which promotes plantlet dormancy. The study findings provide a theoretical basis for future research conducted to modulate tree peony plantlet dormancy.

## 2. Materials and Methods

### 2.1. Materials

*P. suffruticosa* cultivar ‘Daojin’ plantlet roots, stems, leaves, and apical buds were collected, immediately frozen in liquid nitrogen, and stored at −80 °C until used for the full-length transcriptome sequencing analysis. Total RNA was extracted from ‘Daojin’ plantlets for the gene cloning experiment. At approximately 3 months after transplantation, the leaves of the ‘Daojin’ plantlets were injected for the transient transformation and quantitative real-time PCR (qRT-PCR) analysis.

### 2.2. Full-Length Transcriptome Sequencing

Full-length transcriptome sequencing was conducted by Biomarker Technologies Co., Ltd. (Beijing, China). Total RNA was extracted from the root, stem, leaf, and apical bud samples collected from tree peony plantlets. RNA quality was examined using the 2100 Bioanalyzer (Agilent Technologies, Santa Clara, CA, USA). High-quality RNA was used to construct the cDNA library. PacBio long reads were filtered, with redundant sequences removed using CD-HIT-EST. Sequences shorter than 50 bp and those with a sequence accuracy <0.9 were removed. The filtered full-length transcripts were functionally annotated using the NCBI non-redundant protein sequences (NR; https://www.ncbi.nlm.nih.gov/, accessed on 12 November 2023), Swiss-Prot (https://www.uniprot.org/, accessed on 12 November 2023), Gene Ontology (GO; https://www.geneontology.org/, accessed on 12 November 2023), Clusters of Orthologous Genes (COG; https://www.ncbi.nlm.nih.gov/research/cog-project/, accessed on 12 November 2023), EuKaryotic Orthologous Groups (KOG; https://ftp.ncbi.nlm.nih.gov/pub/COG/KOG/, accessed on 12 November 2023), Protein family (Pfam; https://pfam.janelia.org, accessed on 12 November 2023), and Kyoto Encyclopedia of Genes and Genomes (KEGG; https://www.genome.jp/kegg/, accessed on 12 November 2023) databases.

### 2.3. Identification of Tree Peony PsARF Genes

The published genomes [20,21] of *P. suffruticosa* ‘Luoshenxiaochun’ (CNP0000281) and *Paeonia ostii* (CNP0003098) as well as the full-length transcriptome data of *P. suffruticosa* ‘Daojin’ generated by the Institute of Horticulture of Henan Academy of Agricultural Sciences were used to build a local database. Using “auxin response factor” as the keyword, the annotation information for the sequencing results was screened to select *ARF* family members and identify *PsARF* genes containing the Auxin_resp domain. Sequence information for the *PsARF* genes (e.g., nucleic acid sequence, protein sequence, coding sequence, and genome location) was obtained using genome data as the reference.

### 2.4. Phylogenetic Analysis of PsARF Genes

Tree peony PsARF protein sequences were compared with *A. thaliana* AtARF and rice OsARF protein sequences using ClustalX (http://www.clustal.org/, accessed on 20 December 2023). AtARF and OsARF protein sequences were downloaded from online databases (https://www.arabidopsis.org/, accessed on 21 December 2023 and https://www.ricedata.cn/, accessed on 21 December 2023, respectively). A phylogenetic tree was constructed according to the maximum likelihood method using MEGA11 (https://www.megasoftware.net/, accessed on 25 December 2023), with the validation parameter (bootstrap) set to 500. The identified tree peony *ARF* genes were named according to their similarities to *A. thaliana ARF* genes in the phylogenetic tree.

### 2.5. Bioinformatics Analysis of PsARF Genes

On the basis of the *PsARF* sequences, the isoelectric point, molecular weight, and other physicochemical properties of the encoded proteins were predicted using ProtParam (http://web.expasy.org/protparam/, accessed on 2 December 2023). The PsARF domain structures were predicted using NCBI CDD (https://www.ncbi.nlm.nih.gov/Structure/cdd/wrpsb.cgi, accessed on 5 December 2023), whereas conserved motifs were analyzed using MEME 5.5.4 (http://meme-suite.org/tools/meme, accessed on 13 December 2023). The *PsARF* gene structures were analyzed using GSDS 2.0 (http://gsds.cbi.pku.edu.cn, accessed on 29 December 2023).

### 2.6. Gene Cloning and Vector Construction

On the basis of the transcriptome data for dormancy after the rooting process of tree peony plantlets and the phylogenetic relationships with *A. thaliana* genes, *PsARF16a*, which may be related to tree peony plantlet dormancy, was selected for cloning. Total RNA was extracted from tree peony plantlets using the MiniBEST Plant RNA Extraction Kit (TaKaRa, Dalian, China). RNA quality was assessed using the NanoDrop 2000 spectrophotometer (NanoDrop Technologies, Wilmington, DE, USA). High-quality RNA was reverse-transcribed to cDNA using the PrimeScript™ II 1st Strand cDNA Synthesis Kit (TaKaRa). The *PsARF16a* cDNA sequence was amplified by PCR using PrimeSTAR^®^ GXL DNA Polymerase, a high-fidelity Taq enzyme (Baori Medical Biological Technology (Beijing) Co., Ltd.), and the following primers (5′→3′): Fw: GGGGACAAGTTTGTACAAAAAAGCAGGCTTCATGCCTCTGGTGAACTCAAAG; Rv: GGGGACCACTTTGTACAAGAAAGCTGGGTATTACGCAAATATGCTCAAATG. The amplified product was purified and inserted into the pDONR207 vector via a BP reaction (Gateway™ BP Clonase™ II; Thermo Fisher Scientific, Waltham, MA, USA) and then used to construct the overexpression vector pK2GW7 via an LR reaction (Gateway™ LR Clonase™ II; Thermo Fisher Scientific). The resulting recombinant plasmid was inserted into *Agrobacterium tumefaciens AGL1* cells for the subsequent analysis of transient gene expression [22].

### 2.7. Subcellular Localization

To verify that PsARF16a is a transcription factor, we examined its subcellular localization. Specifically, *PsARF16a* was cloned into the p221-GFP vector using the ClonExpress^®^ II One Step Cloning Kit C112-V9.1 (Vazyme, Nanjing, China) to obtain the p221-PsARF16a-GFP recombinant plasmid, which was used for the transfection of *A. thaliana* mesophyll protoplasts according to the PEG method. Protoplasts were extracted and transfected as described by Yoo et al. [23]. After a 16 to 20 h incubation, the subcellular distribution of the recombinant protein was detected using a TCS SP8 fluorescence microscope (Leica, Wiltsburg, Germany) with a 40× magnification objective lens. For the subcellular localization analysis, p221-AtWRKY40-mCherry (Shanghai Plantshop Biotechnology Co., Ltd., Shanghai, China), which was used as a positive control, was co-expressed with PsARF16a-GFP. The GFP and mCherry signals were detected using excitation wavelengths of 488 and 560 nm, respectively, and emission wavelengths of 500–550 and 600–650 nm, respectively. In total, 4 × 10^5^ protoplasts were used for each transfection. Three independent experiments were performed, with similar results. Microscopy images were prepared using Leica Application Suite X software, version 1.4.6, (Leica, Wiltsburg, Germany).

### 2.8. RNA Sequencing and Transcriptome Analysis

The recombinant plasmid carrying *PsARF16a* was injected into the leaves of transplanted tree peony plantlets, whereas MS (Duchefa Biochemie, Haarlem, The Netherlands) liquid medium was injected into the control leaves. For each leaf, the region surrounding the injection site was collected at 24, 36, 48, and 60 h after the injection for the subsequent transcriptome sequencing analysis. Total RNA was extracted using an EASYspin Plus Plant RNA Mini Kit (Beijing Adelai Biological Technology Co., Ltd., Beijing, China). RNA purity and concentration were determined using the NanoDrop ultra-microspectrophotometer 2000 (NanoDrop Technologies, Wilmington, DE, USA). A cDNA library was constructed and sequenced by Beijing Baimike Biotechnology Co., Ltd. The cDNA library quality was evaluated using the 2100 Bioanalyzer (Agilent Technologies), whereas the effective concentration of the cDNA library was determined by qRT-PCR. An Illumina HiSeq 2000 system (Illumina, CA, USA) was used for the paired-end sequencing of the high-quality cDNA library.

The differentially expressed genes (DEGs) among treatments were detected using DEseq [24]. The resulting *p*-values were adjusted using the Benjamini and Hochberg approach for controlling the false-discovery rate (FDR). An FDR < 0.01 and fold-change ≥ 2 were set as the criteria for identifying DEGs. A KEGG pathway enrichment analysis was performed using KOBAS 2.0 [25]. A gene expression heat map was constructed using the Biomarker cloud platform (https://international.biocloud.net/zh/software/tools/list, accessed on 16 February 2024).

### 2.9. Validation by Quantitative Real-Time PCR

To verify the reliability of the transcriptome sequencing data, qRT-PCR analysis was conducted to determine the relative expression levels of 10 DEGs associated with hormone signal transduction and starch and sucrose metabolism. Total RNA was extracted using the MiniBEST Plant RNA Extraction Kit (TaKaRa). The quality of the extracted RNA was evaluated using the NanoDrop 2000 spectrophotometer. High-quality RNA was reverse-transcribed using the PrimeScript™ II 1st Strand cDNA Synthesis Kit (TaKaRa), after which the qRT-PCR analysis was completed using the SYBR Green PCR Master Mix Kit (Takara Bio, Shiga, Japan) and the Bio-Rad CFX instrument (Bio-Rad, Hercules, CA, USA). Relative expression levels were calculated according to the 2^−ΔΔCt^ method, with a tree peony *Pstubulin* gene (Gene.20610::PB-F_transcript_12290/psu.T.00016986.1) serving as an internal control. The expression of all selected genes was analyzed using three replicates. The qRT-PCR data were analyzed using SPSS (SPSS, Inc., Chicago, IL, USA). Information regarding the qRT-PCR primers is listed in Table 1.

## 3. Results

### 3.1. Identification and Physicochemical Characteristics of PsARF Genes

In total, 19 *PsARF* genes were identified on the basis of the genome data and full-length transcriptome data of *Paeonia ostii*, *P. suffruticosa* cultivar ‘Luo shen xiao chun’, and *P. suffruticosa* cultivar ‘Daojin’. The *PsARF* genes in tree peonies were compared with the *AtARF* genes in *A. thaliana* to identify homologs. The tree peony *PsARF* genes were named according to their similarities to *AtARF* genes. Bioinformatics analyses were conducted to obtain basic information regarding the physicochemical properties of *PsARF* genes (Table 2). The PsARF proteins were revealed to contain the B3 and ARF domains, making them true ARF proteins. The Aux/IAA domain was also detected in the PsARF proteins, except for PsARF3, PsARF10, PsARF11b, PsARF16a, PsARF16b, PsARF17, and PsARFx. Sixteen of the *PsARF* genes were distributed on five chromosomes, with chromosome 2 carrying the most (i.e., six *PsARF* genes). The remaining *PsARF* genes were distributed on other chromosomes. The PsARF sequences (367–1005 amino acids) had a predicted molecular weight of 40.17–112.46 kDa, with a theoretical isoelectric point of 4.47–10.11. Most of the PsARF proteins had an isoelectric point less than 7 (i.e., acidic proteins).

### 3.2. Evolutionary Relationships among PsARF Genes

**Figure 1:**
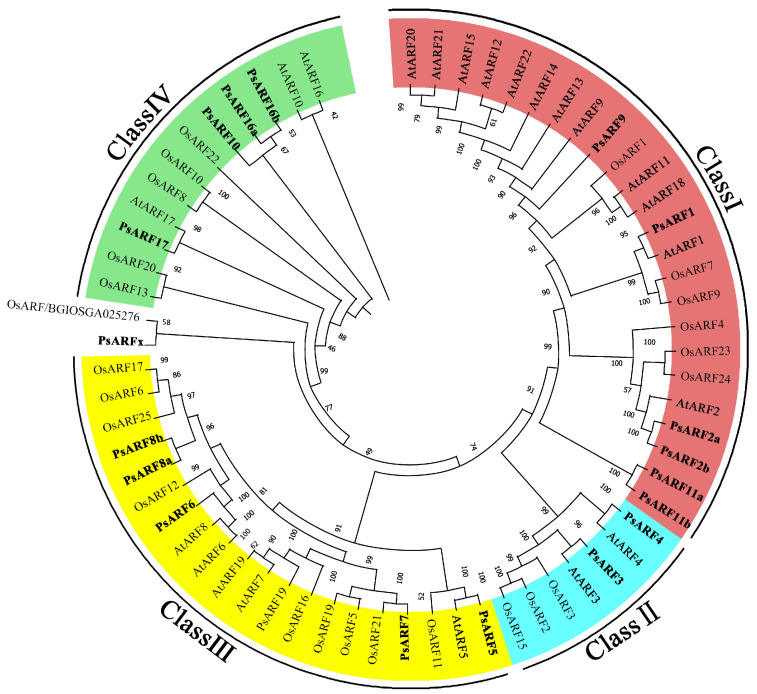
Phylogenetic tree of the *ARF* gene families in tree peonies, *A. thaliana*, and rice. Classes I, II, III, and IV are indicated in red, blue, yellow, and green, respectively. Tree peony *ARF* genes in different classes are in bold.

To reveal the evolutionary relationships of tree peony *PsARF* genes, the PsARF protein sequences and the sequences of 46 ARF proteins in *A. thaliana* and rice were used to construct a phylogenetic tree (Figure 1). Eighteen *PsARF* genes were divided into four classes (Classes I, II, III, and IV), with each class consisting of *AtARF*, *OsARF*, and *PsARF* genes. However, the *PsARF* genes were significantly more closely related to *A. thaliana ARF* genes than to rice *ARF* genes. Similar to the rice gene *OsARFx/BGIOSGA025276*, *PsARFx* was clustered in a branch separate from the other *A. thaliana* and rice *ARF* genes, suggesting the functions of the proteins encoded by these two genes differ from those of the other ARF proteins. Class I was the largest, with 12 *AtARF*, 6 *OsARF*, and 6 *PsARF* genes, but these genes likely evolved relatively independently. Notably, *AtARF12–15* and *AtARF20–22* as well as *PsARF11a* and *PsARF11b* were clustered together, separate from the other *ARF* genes, with no highly homologous sequences detected. Class II included two pairs of *A. thaliana* and tree peony *ARF* genes (i.e., orthologs). In contrast, Classes III and IV included both orthologous and paralogous pairs of *A. thaliana* and tree peony *ARF* genes, with relatively diverse evolutionary patterns.

### 3.3. Conserved Domains and Motifs in PsARF Proteins

**Figure 2:**
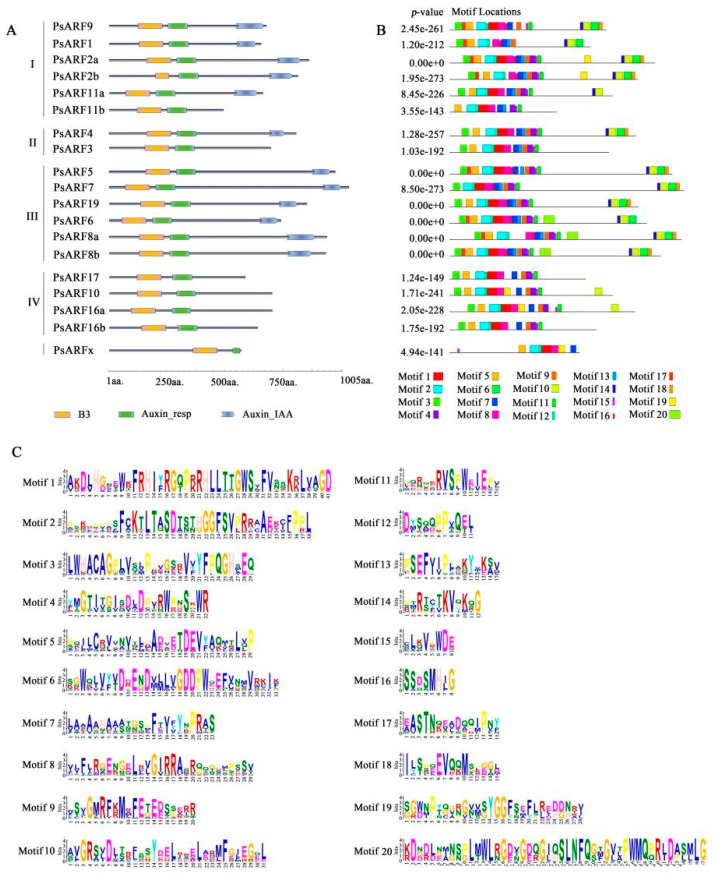
Conserved domains and motifs in the ARF proteins in tree peonies. (**A**) Conserved domains in PsARF proteins. (**B**) Conserved motifs in PsARF proteins. (**C**) Motif logos and amino acid compositions.

According to the conserved domain analysis (Figure 2A), most of the PsARF proteins contained the B3, Auxin_resp, and Aux/IAA3 domains. All of the Class III proteins included the B3, Auxin_resp, and Aux/IAA3 domains. These domains were also detected in the proteins in Class I, except for PsARF11b, which lacked the C-terminal Aux/IAA domain. All of the Class IV proteins lacked the C-terminal Aux/IAA domain but contained the B3 and ARF domains. Similarly, PsARFx also contained only the B3 and Auxin_resp domains. Further analyses of the conserved motifs in PsARF proteins (Figure 2B) revealed differences in the types and number of motifs among PsARF sequences, which may reflect the functional diversity of these proteins. The B3 domain consists of motifs 1, 2, 8, and 12; the Auxin_resp domain consists of motifs 4, 7, 9, 13, and 15; and the Aux/IAA domain consists of motifs 6, 10, and 14. The Auxin_resp domain of PsARFx contained motif 7 but lacked motifs 4, 9, 13, and 15, which may explain why *PsARFx* was included in a separate branch of the phylogenetic tree. The amino acid sequences of each motif (Figure 2C) confirmed that the domains B3, Auxin_resp, and Aux/IAA were highly conserved, as were several amino acid sites in the domains.

### 3.4. PsARF Gene Structures

**Figure 3:**
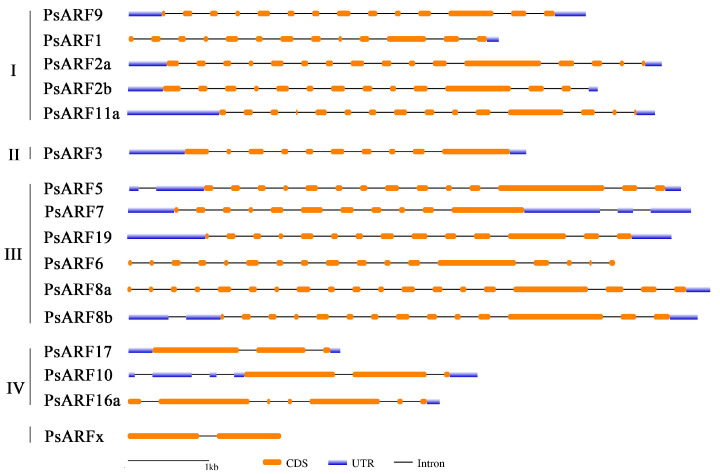
Structural characteristics of *ARF* genes in tree peonies. Orange and blue squares and black lines represent the coding sequence (CDS), untranslated region (UTR), and intron of *PsARF* genes, respectively. The length of 1 kb is indicated at the bottom.

The structures of 16 *PsARF* genes in *Paeonia ostii* [21] were analyzed on the basis of the available *PsARF* gene annotation information (Figure 3). All 16 *PsARF* genes contained exons and introns, with similar exon–intron structures in the *PsARF* genes belonging to the same class. The number of exons varied between *PsARF* genes in different classes. Specifically, Class I genes had 13–16 exons, Class II genes had 10 exons, Class III genes had 11–19 exons, and Class IV genes had 3–7 exons. The *PsARFx* gene contained only two exons and no predicted untranslated region. These results suggest that structural differences among *PsARF* genes may be associated with their functional diversity.

### 3.5. Gene Cloning and Subcellular Localization

**Figure 4:**
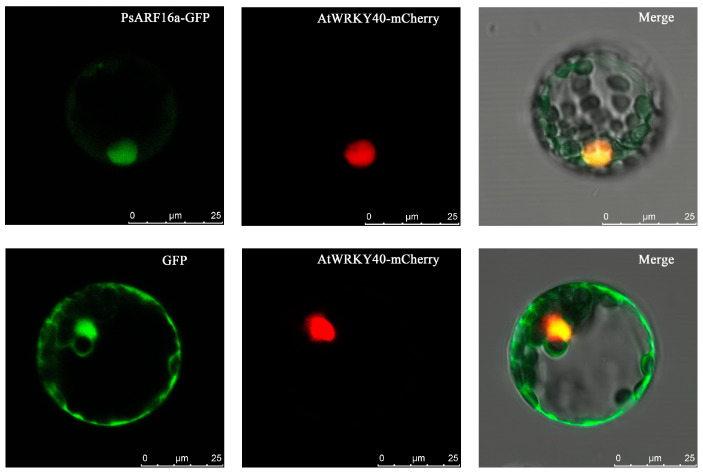
Transient expression and subcellular localization of PsARF16a-GFP. p221-AtWRKY40-mCherry is a nuclear localization marker that was included in the co-transfection of *A. thaliana* mesophyll protoplasts for the transient expression analysis. Fluorescence was detected using a Leica fluorescence microscope. The merged GFP (green) and mCherry (red) signals suggest that PsARF16a was localized in the nucleus. Bar = 25 µm.

Basic information regarding *PsARF16a* is provided in Table 2. Sequencing results indicated that *PsARF16a* (2025 bp) encodes a protein comprising 674 amino acids, which was in accordance with the transcriptome data. During the subcellular localization analysis involving *A. thaliana* mesophyll protoplasts, the fluorescent signals of the PsARF16a-GFP fusion protein and mCherry (positive control) were detected in the nucleus (Figure 4), indicating that PsARF16a is a typical transcription factor that functions mainly in the nucleus.

### 3.6. KEGG Pathway Enrichment Analysis of DEGs

**Figure 5:**
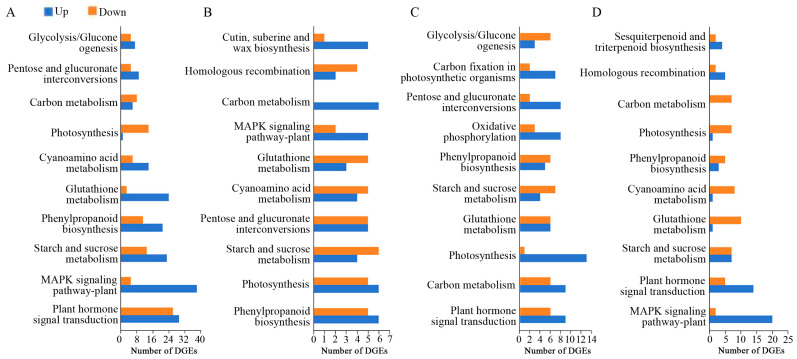
Enriched KEGG pathways among the genes differentially expressed between the treatment and control groups at different post-injection time points. (**A**) Treatment vs. control at 24 h post-injection. (**B**) Treatment vs. control at 36 h post-injection. (**C**) Treatment vs. control at 48 h post-injection. (**D**) Treatment vs. control at 60 h post-injection.

The comparison between the control and samples transiently expressing *PsARF16a* revealed 1644, 605, 881, and 692 DEGs at 24, 36, 48, and 60 h, respectively, with more up-regulated genes than down-regulated genes at all time points. This suggested that many genes in the leaves were involved in a stress response, with significant changes to their expression levels following the transient expression of *PsARF16a*. The number of DEGs was highest at 24 h. Notably, the number of DEGs decreased significantly after 36 h, which indicated that most of metabolic activities in the leaves recovered and stabilized. The significantly enriched KEGG metabolic pathways among the DEGs included the following: plant hormone signal transduction, MAPK signaling pathway-plant, starch and sucrose metabolism, phenylpropanoid biosynthesis, glutathione metabolism, and carbon metabolism (Figure 5). These results may reflect the importance of the regulatory effects of PsARF16a on these metabolic pathways.

### 3.7. DEG Screening and qRT-PCR Verification

**Figure 6:**
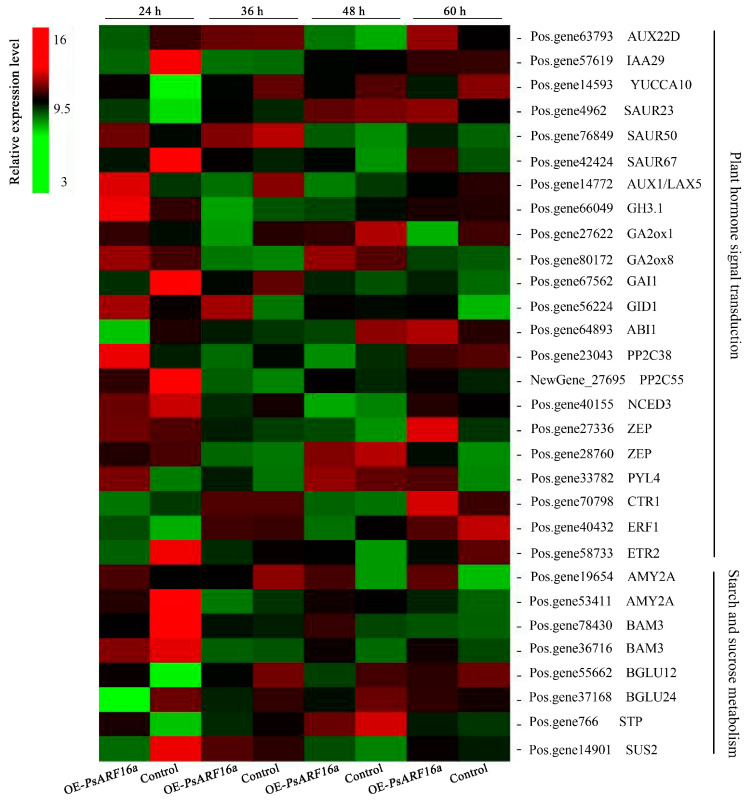
Heat map of differentially expressed genes associated with plant hormone signal transduction as well as starch and sucrose metabolism. According to the transcriptome analysis, the relative expression levels of various genes at different time points were clustered. OE-PsARF16a represents samples transiently overexpressing *PsARF16a*. Red and green indicate high and low relative expression levels, respectively.

Plant dormancy is closely related to endogenous hormones. Moreover, changes to sugar and starch metabolism are an important indicator of plant dormancy. The DEGs identified at different time points suggested the transient expression of *PsARF16a* modulated endogenous hormone signal transduction as well as sugar and starch metabolism. Hence, some key genes involved in endogenous hormone signal transduction or sugar and starch metabolism were screened on the basis of sequence similarity and homology (Figure 6), including IAA signaling pathway-related genes (e.g., *AUX22D*, *IAA29*, *YUCCA10*, *SAUR23*, and *LAX5*), gibberellin (GA) signaling pathway-associated genes (e.g., *GA2ox1*, *GA2ox8*, *GAI1*, and *GID1*), ABA signaling pathway-related genes (e.g., *NCED3*, *ZEP*, *ABI1*, *PP2C38*, *PP2C55*, and *PYL4*), and ethylene signaling pathway-related genes (e.g., *CTR1*, *ERF1*, and *ETR2*). Furthermore, *AMY2A*, *BAM3*, *BGLU12*, *BGLU24*, *STP*, and *SUS2* were the identified genes involved in pathways related to starch and sucrose metabolism.

**Figure 7:**
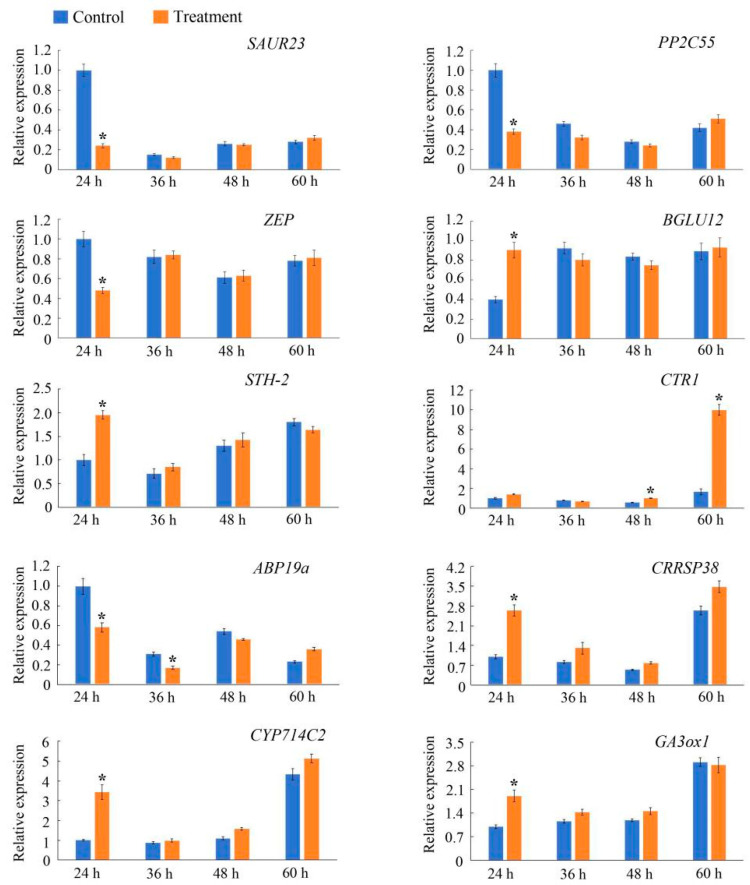
Quantitative real-time PCR analysis of 10 differentially expressed genes in the treatment and control groups at different time points after samples were injected with *PsARF16a*. Error bars represent the standard error of three biological replicates. The asterisks indicate a significant difference (Student’s *t*-test: *p* < 0.05) between samples at different post-injection time points.

To validate the reliability of the RNA-seq results, the following 10 DEGs predominantly associated with plant hormone signal transduction or starch and sucrose metabolism according to the unigene annotations were selected for the qRT-PCR analysis conducted to verify their expression patterns at different time points: *Pos.gene4962*, *NewGene_27695*, *Pos.gene28760*, *Pos.gene55662, Pos.gene41152*, *Pos.gene70798*, *Pos.gene47097*, *Pos.gene25516*, *Pos.gene23350*, and *Pos.gene64704* (Figure 7). The comparison with the control detected significant changes in the expression levels of most of these genes at 24 h after samples were injected with *PsARF16a*, but the expression levels recovered and stabilized after 24 h (i.e., no significant differences with the corresponding control levels). The *Pos.gene4962*, *NewGene_27695*, *NewGene_28760*, and *Pos.gene47097* expression levels were significantly down-regulated at 24 h after the injection with *PsARF16a*, which contrasted with the significantly up-regulated expression of the other genes. The qRT-PCR data were in accordance with the RNA-seq results, indicating that the transcriptome sequencing data were accurate and reliable.

## 4. Discussion

In plants, *ARF* genes encode regulators of multiple critical biological processes related to growth and development [26]. In recent years, fully sequenced genomes of many plant species have been used to identify and analyze *ARF* gene families in different species, including *A. thaliana*, rice, poplar, tomato, corn, soybean, apple, barley, millet, and *Dendrobium officinale* [27]. In the current study, 19 *PsARF* genes were systematically identified by analyzing the genome and transcriptome data of three tree peony cultivars. Although there were similarities in the *PsARF* genes in the examined cultivars, there were also some differences. For example, *PsARF4* was detected only in the genome of *P. suffruticosa*, whereas *PsARF17* was exclusive to *P. ostii*, and *PsARF16b* was unique to ‘Daojin’. Phylogenetic analyses are important for elucidating gene family functions. Because of the relatively limited molecular-biology-based research on tree peonies, phylogenetic trees are critical for classifying tree peony gene functions. The 19 *PsARF* genes identified in this study were divided into four classes, which was consistent with the results of related research on pears [28] and grapes [29]. According to the constructed phylogenetic tree, *PsARFx* and the rice gene *OsARFx/BGIOSGA025276* were distantly related to the other *ARF* genes in tree peonies, *A. thaliana*, and rice. Additionally, the Auxin_resp domain of PsARFx was incomplete (i.e., missing motifs 4, 9, 13, and 15), suggestive of incomplete gene transcription. Moreover, only two exons were detected in *PsARFx*, which may help to explain why this gene belonged to a separate branch of the phylogenetic tree. Compared with other PsARF proteins, PsARFx may have a different function because of its incomplete structure. Most of the examined PsARF proteins contained three conserved domains. The PsARF proteins in the same class had similar conserved domains and motifs. Similarly, the *PsARF* genes belonging to the same class were similar in terms of their exon–intron structures. These results reflect the reliability of the phylogenetic tree constructed on the basis of a multiple sequence alignment. Interestingly, the Class IV PsARF proteins lacked the Aux/IAA (CTD) domain. A previous study showed ARF activators and Aux/IAA suppressors can inhibit gene expression via the dimerization of the CTD domain [30]. The functions of ARF proteins lacking the CTD domain may not involve auxin signaling pathways [26], ultimately resulting in functional diversification. The *PsARF* genes in Class IV had fewer exons than the *PsARF* genes in the other classes, which was consistent with the findings of earlier related studies on apples [31] and pears [28]. Although the tree peony genome is larger than the *A. thaliana* genome, the *PsARF* gene family is smaller than the *AtARF* gene family, mainly because the tree peony has considerably fewer Class I genes than *A. thaliana*. This phenomenon was also observed in other plant species, including barley [32], apple [31], sweet orange [33], *Eucalyptus grandis* [34], and litchi [35]. Hence, similar to these species, the tree peony may have lost some *PsARF* genes during its evolution.

Earlier studies showed *ARF* genes are important for several processes related to plant growth and development, including flower and cotyledon formation, plant embryogenesis, leaf organ senescence, fruit maturation, and dormancy [36,37]. In the present study, our analysis of 65 *ARF* genes from *A. thaliana*, rice, and tree peonies showed that *PsARF10* and *PsARF16* are highly homologous to *AtARF10* and *AtARF16*. Both AtARF10 and AtARF16, which are essential for maintaining *ABI3* expression in seeds, can activate *ABI3* transcription and promote seed dormancy [8]. Therefore, PsARF10 and PsARF16 may have similar functions and regulate tree peony plantlet dormancy. Dormancy induction and release are regulated by various signaling molecules, including sugars and plant hormones. Specifically, hormones act as the first signal in the dormancy-related signal transduction pathway, inhibiting or activating the activities of intracellular protein factors and enzymes by binding to receptors on the target cell membrane, thereby regulating gene expression and metabolism [38]. Endogenous ABA, GA, auxin (e.g., IAA), and zeanoside are the four major hormones that regulate plant dormancy and germination [39]. Of these hormones, ABA induces plant dormancy. Decreases in the GA content inhibit plant growth but do not induce dormancy [40]. Auxin is the most important regulator of bud dormancy, with IAA modulating P450 monooxygenase gene expression, while also controlling the ABA content, to induce bud dormancy [41]. In the current study, the significantly enriched pathways among the DEGs in *PsARF16a*-overexpressing tree peony plantlet leaves were related to plant hormone signal transduction as well as starch and sugar metabolism. The expression levels of most DEGs changed significantly at 24 h after *PsARF16a* was injected into the leaves, after which they recovered and stabilized, with no significant difference from the corresponding control level. Zeaxanthin epoxidase (ZEP) and 9-cis-epoxycarotenoid dioxygenase (NCED) are two enzymes that regulate ABA biosynthesis [42]. In the current study, the overexpression of *PsARF16a* may have promoted the transcription of *ABI*, and *ABI1* expression was significantly up-regulated at 60 h, which activated the ABA signal transduction pathway, resulting in the up-regulated expression of *NCED3* at 36 and 60 h. In addition, *ZEP* expression was significantly down-regulated at 24 h, whereas ABA synthesis increased. Furthermore, the expression of the ABA receptor gene *PYL* was significantly up-regulated. Earlier research showed that PYL/PYR can initiate ABA signal transduction by inhibiting the activity of the protein phosphatase PP2C [43]. The PP2C protein HONSU is a key factor regulating the ABA/GA balance, which influences seed dormancy [44]. In our study, the *PYL* expression level was significantly up-regulated, which may have inhibited PP2C activities and significantly down-regulated *PP2C* expression at 24 h, thereby initiating ABA signal transduction and altering the ABA/GA balance. Consequently, the genes involved in GA metabolism were activated. The expression levels of *GA2ox1* (GA synthase) and *GID1* (GA receptor factor) were significantly up-regulated at 24 h. In tree peony plantlets, the GA_3_ content decreases significantly after rooting, whereas *GID1* expression is significantly up-regulated [5]. Changes in carbohydrate content are important indicators of plant dormancy and germination, with the conversion of sugar and starch directly affecting the dormancy process. Dormancy release is the result of a shift in glucose metabolism from the glycolytic/tricarboxylic acid cycle to the pentose phosphate pathway [45]. During dormancy release and germination, starch hydrolysis is accelerated, with the associated increase in soluble sugar contents (sucrose, fructose, and glucose) enhancing the activities of a series of starch-degrading enzymes and increasing the expression of genes encoding the relevant enzymes [46,47]. However, the opposite changes occur during dormancy induction. Specifically, various metabolic activities are weakened or completely inhibited, energy consumption decreases substantially, and energy storage increases after plants enter dormancy. In our study, the expression levels of some key genes associated with starch and sugar metabolism were significantly affected by the overexpression of *PsARF16a*. For example, *AMY2A*, *BAM3*, and *BGLU* expression levels were significantly up-regulated at 24 h, which was in contrast to the significant decrease in the expression of *STP* and *SUS2*. A previous study indicated *BGLU* is a key gene in the sugar synthesis pathway, and its expression is closely associated with sugar contents [48]. These results imply that *PsARF16a* overexpression promoted leaf dormancy, with weakened metabolic activities, accelerated starch synthesis, and the gradual conversion of sugar into starch. On the basis of the study findings, we speculate that after plantlets were transferred to the rooting medium, the accumulation of a large amount of auxin enhanced the response to *PsARF16a* expression, which activated *ABI* transcription and induced the expression of some key genes that increased ABA synthesis and decreased GA synthesis, ultimately leading to imbalanced ABA and GA contents and plantlet dormancy.

## 5. Conclusions

In this study, 19 *PsARF* genes in three tree peony cultivars were identified and analyzed in terms of their physicochemical properties, gene structures, and protein structures. Our analysis of transient expression in tree peony leaves revealed the importance of *PsARF16a* for regulating ABA signal transduction and ABA synthesis. Moreover, PsARF16a may be a critical regulator of tree peony plantlet dormancy. The study data may form the basis of future investigations conducted to functionally characterize *PsARF* genes and further clarify the molecular mechanism underlying tree peony plantlet dormancy.

## Figures and Tables

**Table 1:** Primers used for quantitative real-time PCR analyses of differentially expressed genes.

Gene Name	Gene ID	Forward Primers (5′→3′)	Reverse Primers (5′→3′)
*SAUR23*	Pos.gene4962	GCTTACCTCAACCATCCTTCA	CTTGGCATTAGCAATCATCG
*PP2C55*	NewGene_27695	CGTTGGAAGGTATTAGTGGAGG	GGAGAGCCTGACCATCAAAG
*ZEP*	Pos.gene28760	GATACATTCACACCTGCGGT	TTCTTCCTCACCTTTGACCA
*BGLU12*	Pos.gene55662	TGTTACCCTGTTTCACTGGG	CCTTGTAGCAAAGTTCCGC
*STH-2*	Pos.gene41152	AACCAAGTTTGCCCAAGG	GCTCAACGCCATCTTTAGC
*CTR1*	Pos.gene70798	TGTGATGTGGGAGATGGTTAC	CTGCCAACAACGCTTCAT
*ABP19a*	Pos.gene47097	TGACTTCTGTGTGGCTAACC	TGTTTGAGGTATTCCCAGC
*CRRSP38*	Pos.gene25516	TCACTGCCAACAACCCTTAC	GACACAGAGCAAGCCCATT
*CYP714C2*	Pos.gene23350	GCACACAGACCCAGAAGTATG	AAGCGGGCGAGTGATTAT
*GA3ox1*	Pos.gene64704	TTTGGGTGATGCGTCTCA	ACCAGGAATAGGAACAGCCC
*Pstubulin*	Gene.20610::PB-F_transcript_12290/psu.T.00016986.1	CAAGTGTTTGTGACATTCCTCC	CCATTTCATCCATACCTTCTCC

**Table 2:** Basic information regarding the *ARF* gene family in tree peonies.

Gene Name	Gene ID	Gene ID	Gene ID	Protein Length/aa	pI	Molecular Weight/kDa	Distribution on Chromosomes	Domain
*PsARF1*	Gene.131018::PB-F_transcript_97250	Pos.gene22084	psu.T.00012958.1	652, 604, 369	7.73, 7.32, 7.79	73.06, 67.99, 41.35	2	B3, ARF, Aux/IAA
*PsARF2a*	Gene.7512::PB-F_transcript_2941	Pos.gene36395	/	854, 880, /	6.49, 6.47, /	94.96, 98.06, /	2	B3, ARF, Aux/IAA
*PsARF2b*	/	Pos.gene33192	psu.T.00031102.1	/, 807, 726	/, 6.80, 7.86	/, 89.92, 80.67	5	B3, ARF, Aux/IAA
*PsARF3*	Gene.138423::PB-F_transcript_101725	Pos.gene54551	psu.T.00033043.1	684, 684, 684	6.66, 6.66, 6.66	74.99, 74.99, 74.99	5	B3, ARF
*PsARF4*	/	/	psu.T.00008733.1	/, /, 800	/, /, 6.01	/, /, 88.77	/	B3, ARF, Aux/IAA
*PsARF5*	Gene.99507::PB-F_transcript_80590	Pos.gene48813	/	946, 954, /	5.13, 5.26, /	104.44, 105.44, /	2	B3, ARF, Aux/IAA
*PsARF6*	Gene.81267::PB-F_transcript_71012	Pos.gene59490	/	720, 843, /	6.00, 6.16, /	80.89, 94.80, /	4	B3, ARF, Aux/IAA
*PsARF7*	Gene.71676::PB-F_transcript_66112	Pos.gene80351	psu.T.00013165.1	788, 714, 1005	6.17, 7.93, 6.99	87.77, 80.67, 112.46	4	B3, ARF, Aux/IAA
*PsARF8a*	Gene.5886::PB-F_transcript_2127	Pos.gene47864	psu.T.00026218.1	889, 993, 847	6.52, 6.90, 6.66	98.60, 109.83, 93.84	1	B3, ARF, Aux/IAA
*PsARF8b*	Gene.3745::PB-F_transcript_1154	Pos.gene50509	psu.T.00005392.1	917, 905, 477	6.76, 6.68, 9.74	101.69, 100.42, 52.69	5	B3, ARF, Aux/IAA
*PsARF9*	Gene.9265::PB-F_transcript_3919	Pos.gene67379	psu.T.00003487.1	673, 673, 673	6.92, 6.92, 6.92	75.08, 75.08, 75.08	1	B3, ARF, Aux/IAA
*PsARF10*	Gene.155669::PB-F_transcript_110701	Pos.gene37675	/	699, 699, /	6.86, 6.86, /	77.39, 77.37, /	2	B3, ARF
*PsARF11a*	Gene.108016::PB-F_transcript_85046	Pos.gene39948	psu.T.00005912.1	663, 700, 709	6.78, 7.00, 7.10	73.83, 77.28, 79.07	3	B3, ARF, Aux/IAA
*PsARF11b*	Gene.112109::PB-F_transcript_87131	/	psu.T.00019060.1	464, /, 461	9.61, /, 10.11	52.25, /, 52.50	/	B3, ARF
*PsARF16a*	Gene.163854::PB-F_transcript_115372	Pos.gene62772	psu.T.00016095.1	674, 794, 601	8.00, 7.65, 8.05	74.51, 87.92, 66.66	1	B3, ARF
*PsARF16b*	Gene.12697::PB-F_transcript_6183	/	/	630, /, /	8.60, /, /	69.68, /, /	/	B3, ARF
*PsARF17*	/	Pos.gene21092	/	/, 584, /	/, 4.98, /	/, 65.25, /	2	B3, ARF
*PsARF19*	Gene.119605::PB-F_transcript_91086	Pos.gene73404	/	367, 810, /	4.47, 5.53, /	40.17, 89.34, /	3	B3, ARF, Aux/IAA
*PsARFx*	/	Pos.gene73377	psu.T.00012042.1	/, 557, 444	/, 7.35, 6.20	/, 62.60, 50.54	2	B3, ARF

## Data Availability

The data are included within the article.
